# Extraction of flavanones from immature *Citrus unshiu* pomace: process optimization and antioxidant evaluation

**DOI:** 10.1038/s41598-020-76965-8

**Published:** 2020-11-17

**Authors:** Dong-Shin Kim, Sang-Bin Lim

**Affiliations:** Department of Food Bioengineering, Jeju National University, Jeju, 63243 Republic of Korea

**Keywords:** Nutrition, Nutritional supplements

## Abstract

Dietary guidelines recommend the consumption of flavonoid-rich extracts for several health benefits. Although immature *Citrus unshiu* pomace (ICUP) contains high levels of flavanone glycosides, many studies have concentrated on the optimization of flavonoid extraction from mature citrus peels. Therefore, we developed an optimized extraction method for hesperidin and narirutin from ICUP, and evaluated their antioxidant activities using ten different assay methods. The extraction conditions for the highest flavonoid yields based on a response surface methodology were 80.3 °C, 58.4% (ethanol concentration), 40 mL/g (solvent/feed), and 30 min, where the hesperidin and narirutin yields were 66.6% and 82.3%, respectively. The number of extractions was also optimized as two extraction steps, where the hesperidin and narirutin yields were 92.1% and 97.2%, respectively. Ethanol was more effective than methanol and acetone. The ethanol extract showed high scavenging activities against reactive oxygen species but relatively low scavenging activities for nitrogen radicals and reactive nitrogen species. The antioxidant activities showed a higher correlation with hesperidin content than narirutin content in the extracts. This study confirms the potential of an optimized method for producing antioxidant-rich extracts for the functional food and nutraceutical industries.

## Introduction

Oxidative stress is usually caused by reactive oxygen species (ROS) and reactive nitrogen species (RNS), which can attack healthy cells, causing them to lose function and structure^1^. More than 100 diseases are reportedly associated with oxidative stress, including Alzheimer’s disease, atherosclerosis, and cancer^1,2^. ROS and RNS can be eliminated by natural antioxidants, and various in vitro methods that measure the antioxidant activities of natural compounds have been reported^3–5^. The antioxidant activity of natural compounds may vary depending on the antioxidant mechanism (hydrogen atom transfer and electron transfer), the structure of the antioxidant, and the radicals; therefore, various assay methods should be applied to comprehensively test the antioxidant activity of diverse natural antioxidants^3,5,6^.

Flavonoids are secondary metabolites of natural plants, which reportedly have many health benefits in humans. Citrus species are a rich source of flavanone glycosides such as hesperidin and narirutin, which have antioxidant, anticancer, anti-inflammatory, and antiobesity activities^7–9^. In particular, immature *Citrus unshiu* fruit is a good source of flavonoid supplements, because it contains higher levels of flavanones than the mature fruit^10^.

Currently, the flavonoid extraction from citrus varieties has attracted considerable scientific and industrial attention, and various methods were reported in the literature for the recovery of phenolic compounds mainly from mature citrus peels^11–18^. The conventional solvent extraction methods are mainly used, such as maceration and refluxing, for the industrial applications. Other methods are also used, such as microwave-assisted, ultrasound-assisted, subcritical water, supercritical fluid, and high pressure extractions. Those novel extractions provide advantages over extraction yields, solvent consumption, and extraction time compared to the conventional extraction methods. However, these methods have limited application fields, and further research is required to improve the understanding of extraction mechanisms, design, and scale-up for industrial applications^12–14,16,18^ .

Maceration has been used as an inexpensive technique in the extraction of phenolic compounds. The refluxing takes less solvent and extraction time, and is more efficient than maceration^18^. Most of the studies have shown that methanol extraction resulted in a higher yield of citrus flavonoids^12–16,19^. However, methanol is avoided in food industrial applications because it is toxic. Ethanol is considered “Generally Recognized As Safe” (GRAS) solvent by the US Food and Drug Administration and is the most recommended solvent during the food industrial extraction processes^18,20^, therefore it was tested in this study.

Several authors studied the effect of ethanol extraction on the citrus flavonoid recovery. Lee et al.^21^ optimized the hesperidin extraction from mature *C. unshiu* peel (extraction conditions: sample/solvent 1 g/10 mL, ethanol 50, 100%, temperature 30, 60, 90 °C, time 2, 13, 24 h); the optimal condition was 71.5 ºC, 59.0%, and 12.4 h, where the hesperidin yield was 287.8 μg per 5 mg extract. In this case, the extraction time was too long (12.4 h) despite the small sample size (1 g) of *C. unshiu* peel. Li et al.^11^ extracted total phenolics from the peels of lemons, grapefruit, mandarin (*C. reticulata* cv. Ellendale), and sweet orange (extraction conditions: 2 g/16 mL, ethanol 20, 50, 72, 85, 95%, temperature 19, 37, 50, 65, 80 °C, single (3 h) and double (2 × 1.5 h) extractions); the yield of total phenolics increased as the ethanol concentration increased to 85% and as the extraction temperature increased with an exception at 37 °C, and a single-extraction resulted in higher yield than a double-extraction. In this case, they performed only a one-variable-at-a-time experiment using citrus peels, did not conduct experiments at temperatures above 80 °C, and did not measure the composition of hesperidin and narirutin in the extracts. Garcia-Castello et al.^16^ extracted flavonoids from grapefruit wastes (pulp, albedo and flavedo) (extraction conditions: 5 g/ 40 mL, ethanol 20, 50, 80, 100%, temperature 25, 34, 48, 61, 70 °C, time 30, 130, 270, 413, 510 min); the optimum extraction condition was 69 °C, 30%, and 190 min, where total phenolic content was 56.0 mg GAE/g DW and total antioxidant activity was 23.5 mmol trolox/g DW. In this case, they did not conduct experiments at temperatures above 70 °C, the extraction time was too long (190 min), and the optimum ethanol concentration was low (30%). Assefa et al.^17^ extracted flavonoids from yuzu peel (extraction conditions: ethanol 20, 40, 60, 80, 100%, temperature 15, 30, 45, 60, 75 °C, time 30, 60, 90, 120, 150 min, solvent/sample 10, 20, 30, 40, 50 mL/g); the optimized condition was 43.8 °C, 65.5%, 119.6 min, and 37.1 mL/g, where the yields of hesperidin, naringin, and phloretin were 337.2, 244.9, and 43.9 mg/g DW, respectively. In this case, they did not conduct experiments at temperatures above 75 °C, the extraction time was too long (119 min) despite the small sample size (1 g) of yuzu peel, and the optimum temperature was low (43.8 °C). Safdar et al.^22^ extracted polyphenols from kinnow (*C. reticulate* L.) peel (extraction conditions: 5 g/75 mL, ethanol 50, 80, 100%, 40 °C, 20 h, shaking); 80% ethanol yielded 92.9 and 65.2 μg/g dry sample of hesperidin and ferulic acid, respectively. In this case, they performed only a one-variable-at-a-time experiment using kinnow peel and low temperature (40 °C) was used for long extraction time (20 h).

Therefore, in this study immature citrus pomace which contains higher levels of flavanones and has a different sample matrix was used as a test material unlike most previous studies using mature citrus peels. The extraction temperature was tested up to 90 °C to determine the inflection point of the maximum yield of flavanone glycosides, and the extraction time was shortened as much as possible. In addition, the ratio of sample and solvent was extensively tested, and the number of extracts was also optimized by repeated experiments to overcome the equilibrium limit, which corresponds to the exhaustion of flavonoids in the sample matrix^14^.

There have been a few attempts to analyze flavonoid content in immature citrus fruits^10,23,24^, but no optimized method has been developed for recovering flavonoids from immature citrus fruits. To develop flavonoid-rich supplements using immature citrus fruits, the extraction method should be optimized for the highest recovery of index compounds. Extraction parameters such as the solvent nature and concentration, temperature, solvent-to-feed (S/F) ratio, extraction time, and number of extractions influence the yields of phenolic compounds^11–14,16–19^.

We developed an optimized extraction method for maximum recovery of flavanone glycosides such as hesperidin and narirutin from immature *Citrus unshiu* pomace (ICUP), and evaluated the antioxidant activities of the extracts. First, the effects of ethanol concentration, extraction temperature, S/F ratio, and extraction time on the yields of hesperidin and narirutin from ICUP were evaluated individually through single-factor experiments. The optimum extraction conditions were then investigated to maximize the extraction yields using a response surface methodology (RSM). The number of extractions and solvent type were further evaluated under the optimum extraction conditions. In addition, 10 types of antioxidant activity assays (against nitrogen radicals, RNS, ROS, and reducing capacities) were applied to comprehensively evaluate the antioxidant activities of the extracts.

## Materials and methods

### Sample preparation

Immature *C*. *unshiu* Markovich fruits (Table 1) were obtained from a farm in Jeju, Korea. The fresh fruits were rinsed, cut into quarters, and ground using a blender (Shinil Industrial Co., Ltd., Chungcheongnam-do, Korea) for 90 s. The homogenates were pressed using a screw type juice extractor (Green Power, Daejeon, Korea), and the pomace was separated from the juice. The juice was filtered using cotton filter (pore size: 1 mm, Dae Han Medical Supply Co., Ltd., Chungcheongbuk-do, Korea) to recover the fine particles, which then were combined with the pomace. The mixed pomace was freeze-dried, crushed into powder (14–50 mesh), and stored in a freezer at − 20 °C.

**Table 1:**
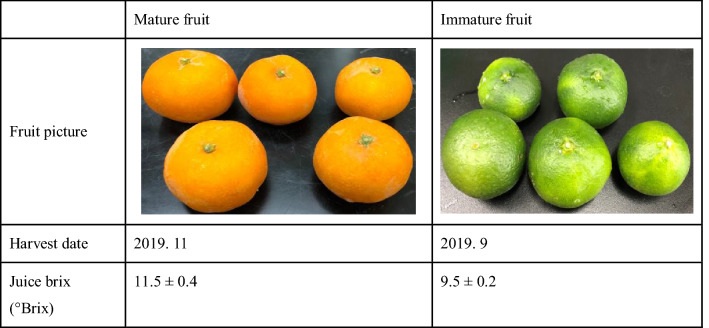
Comparison of mature and immature fruits of *Citrus unshiu*.

### Chemicals and reagents

The list of chemicals and reagents are provided in the Supplementary Information S1.

### Determination of flavonoid contents in mature and immature citrus fruits and pomace

Ten pieces of mature or immature *C*. *unshiu* whole fruits (Table 1) (about 700 g) were ground using a blender (Shinil Industrial Co., Ltd.). Then 5 g pulverized fruits was added to 30 mL methanol and stirred for 30 min at room temperature. The mixture was centrifuged (3000×*g*, 10 min), the supernatant was separated, and the residue was extracted four more times until all flavonoids were recovered quantitatively. The combined extract was concentrated, adjusted to a specific volume with methanol, and filtrated using a syringe filter (0.45 μm) before high-performance liquid chromatography (HPLC) analyses. The water content (%) of each whole fruit was measured after drying in the oven at 105 °C. The content of flavonoids in ICUP were also determined using the same extraction method.

### Extraction procedure of immature *C. unshiu* pomace

The dried ICUP (1 g) was transferred to a flask. The preheated extraction solvent was added to the sample, and then a condenser (glass tube, 6.2 mm ID × 400 mm length) was connected to the flask to collect a vaporized solvent. The extraction was performed in a shaking water bath (120 rpm) at the desired experimental conditions. The extraction mixture was filtrated and adjusted to a specific volume with an extraction solvent. All extracts were filtrated using a syringe filter (0.45 μm) before HPLC analyses.

### Single-factor experiments

The effects of extraction parameters (ethanol concentration, temperature, S/F ratio, and extraction time) on the yields of hesperidin and narirutin from ICUP were individually evaluated. The ethanol concentration was evaluated at 20%, 40%, 60%, 80%, and 100% (v/v); temperatures of 25 °C, 35 °C, 45 °C, 60 °C, 75 °C, 85 °C, and 90 °C; S/F ratios of 20, 30, 40, 50, 60, and 70 mL/g dry sample; and extraction times of 10, 20, 30, 40, 50, and 60 min.

### Response surface design

In a single-factor experiment, three independent variables and ranges were used to evaluate the relationships between the independent variables and responses through RSM: temperature (X_1_: 60 °C, 75 °C, and 90 °C), ethanol concentration (X_2_: 40%, 60%, and 80% (v/v)), and S/F ratio (X_3_: 20, 30, and 40 mL/g dry sample), with an extraction time of 30 min. The Box–Behnken design (BBD) with three factors and levels consisted of 12 runs with independent variables (runs 1–12) and five replicates at the central point (runs 13–17). The following second-order polynomial equation was used to describe the relationship between the independent variables and the responses:

1 $$ Y = \beta_{0 } + \mathop \sum \limits_{i = 1}^{3} \beta_{i} X_{i} + \mathop \sum \limits_{i = 1}^{3} \beta_{ii} X_{ii}^{2} + \mathop \sum \limits_{i = 1}^{2} \mathop \sum \limits_{j = i + 1}^{3} \beta_{ij} X_{ii} X_{ij} $$

where *Y* is the extraction yield, *X*’s are extraction parameters, and *β*’s are coefficients. DESIGN-EXPERT 11.0 software (Trial version, Stat-Ease Inc., Minneapolis, MN, USA) was used to fit the experimental data to the model equation.

### Number of extractions

To determine the effect of the number of extractions on the yields of hesperidin and narirutin from ICUP, extractions were done three times under the optimum conditions for ethanol (80.3 °C, 58.4%, and 40 mL/g dry sample for 30 min).

### Extraction solvents

The effects of different extraction solvents on the yields of hesperidin and narirutin from ICUP were evaluated using methanol and acetone under the optimum conditions obtained using ethanol (80.3 °C, 58.4%, and 40 mL/g dry sample for 30 min).

### HPLC analyses

The contents of hesperidin and narirutin in the extracts were quantified as described by Kim and Lim^25^. The hesperidin and narirutin profiles in the extracts were analyzed using Alliance 2965 HPLC (Waters Corp., Milford, MA, USA). An Inertsil ODS-3 V column (4.6 mm × 250 mm, 5 μm particle size, GL Science, Tokyo, Japan) was used for the separation of each flavonoid. Acetic acid (0.5%) in water (phase A) and acetonitrile (phase B) were utilized for the mobile phase. The solvent flow rate was 1.0 mL/min, using gradients of B: 0 min 15%, 8 min 25%, 15 min 25%, 35 min 65%, 37 min 65%, and 39 min 15%. The hesperidin and narirutin were detected at 290 nm. Each flavonoid was identified by comparing with its retention time and UV–visible spectrum to those of the standard compound. Calibration curves were constructed between absorbance at 290 nm and the concentrations of hesperidin (25–200 mg/mL) and narirutin (10–80 mg/mL) in methanol; the correlation coefficients (*R*^2^) were 0.9996 and 0.9994, respectively.

### Antioxidant activity

Nitrogen radical scavenging activities (DPPH and ABTS) were determined. DPPH radical scavenging activity was evaluated as described by Ye et al.^26^. Each extract (0.1 mL) was mixed with 2.0 mL of a DPPH solution (0.2 mM) and left for 30 min. The absorbance was determined at 517 nm. ABTS radical scavenging activity was also evaluated as described by Yi et al.^27^. Each extract (0.02 mL) was mixed with 0.98 mL of an ABTS solution (0.2 mM) and left for 30 min at 30 °C. The absorbance was evaluated at 750 nm.

RNS scavenging activities (nitrite and nitric oxide) were measured. Nitrite scavenging activity was evaluated as described by Kim and Lim^25^. Each extract (0.2 mL) was mixed with 0.1 mL of sodium nitrite (1 mM) and 0.7 mL of HCl (0.1 N) and left for 60 min at 37 °C. Then, added with acetic acid (0.5 mL) and Griess reagent (0.4 mL) and left for 15 min. The absorbance was determined at 540 nm. Nitric oxide radical scavenging activity was also evaluated as described by Soares et al.^28^. Each extract (0.25 mL) was mixed with 0.5 mL of sodium nitroprusside (10 mM) and left for 3 h at 25 °C under the light. Then, added with 0.75 mL of modified Griess reagent (2% sulphanilamide + 0.2% N-(1-naphthyl) ethylenediamine dihydrochloride in 5% phosphoric acid). The absorbance was measured at 540 nm.

ROS scavenging activities (ORAC, hydroxyl radical, superoxide anion radical, hydrogen peroxide) were determined. ORAC was measured as the methods described by Ye et al.^26^ and Kim and Lim^25^. Each extract (0.025 mL) was mixed with 0.15 mL of fluorescein sodium salt solution (78 nM), left for 15 min at 37 °C, and added with 0.025 mL of AAPH (250 mM). The fluorescence was determined at 37 °C every 3 min for 2 h (excitation, 485 nm; emission, 535 nm). Hydroxyl radical scavenging activity was measured as the method described by Sannasimuthu et al.^29^. Each extract (0.2 mL) was mixed with 0.2 mL of 1,10-phenanthroline (5 mM), 0.2 mL of EDTA (15 mM), and 0.2 mL of FeSO_4_ (5 mM). The reaction was started with an addition of 0.2 mL of H_2_O_2_ (0.03%) at 37 °C for 60 min. The absorbance was evaluated at 536 nm. Superoxide anion radical scavenging activity was measured as described by Kuda et al.^30^. Each extract (0.1 mL) was mixed with 0.1 mL of (0.156 mM) and 1 mL of (0.468 mM) NADH. Then, added with 0.1 mL of phenazine methosulphate (0.06 mM) and left for 5 min. The absorbance was evaluated at 560 nm. Hydrogen peroxide scavenging activity was evaluated as described by Oh and Shahidi^31^. Each extract (0.4 mL) was mixed with 0.6 mL of H_2_O_2_ (40 mM) and left for 40 min at 30 °C. The absorbance was evaluated at 230 nm.

Reducing abilities (reducing power and FRAP) were determined. Reducing power was measured as described by Kim and Lim^25^. Each extract (0.1 mL) was mixed with 0.5 mL of phosphate buffer (0.2 M) and 0.5 mL of potassium ferricyanide (0.1%) and left for 20 min at 50 °C. Then, added with 0.5 mL of trichloroacetic acid (10%) and 0.5 mL of ferric chloride (0.1%) and left for 5 min. The absorbance was determined at 700 nm. Ferric reducing antioxidant power (FRAP) was evaluated as the method described by Ye et al.^26^. The FRAP solution was prepared with 300 mM acetate buffer, 20 mM FeCl_3_, and 10 mM TPTZ (1:1:10 (v/v)). FRAP reagent (3 mL) and distilled water (0.3 mL) were added to 0.2 mL of extract and left for 30 min at 37 °C. The absorbance was observed at 595 nm. All antioxidant activities were expressed as mg Trolox equivalents (mg TE)/g dry sample.

### Statistical analyses

The differences between experimental data were analyzed using Duncan’s multiple range test (*p* < 0.05). The validity between the predicted and experimental data was analyzed using the Student’s *t*-test. Pearson correlation coefficients between antioxidant activities and flavonoid contents in the extracts were also calculated using a bivariate correlation analysis. All statistical analyses were performed using SPSS software (ver. 24.0; SPSS Inc., Chicago, IL, USA).

## Results and discussion

### Flavonoid compositions of mature and immature *C. unshiu* fruits and pomace

The flavonoid compositions of mature and immature *C*. *unshiu* fruits and pomace were determined (Table 2). Hesperidin and narirutin are the major flavonoids (flavanone glycosides), and sinensetin, nobiletin, 3,5,6,7,8,3′,4′-heptamethoxyflavone, and tangeretin are the minor flavonoids (polymethoxylated flavones) in *C*. *unshiu*. The contents of hesperidin and narirutin were 2.32- and 2.34-fold higher in immature than mature *C*. *unshiu* fruits, respectively. Therefore, immature *C*. *unshiu* fruits may be a good candidate as a flavonoid supplement for several health benefits. The content of hesperidin in immature *C*. *unshiu* pomace was the same as that in immature fruits, but that of narirutin in immature pomace was lower than that in immature fruits because it was transferred to the juice when the fruits was squeezed using an extractor due to the more polar property of narirutin compared with hesperidin^32^.

**Table 2:** Flavonoid compositions of mature and immature *C*. *unshiu* fruits and pomace.

Flavonoid	Content (μg/g dry sample)		
	Mature fruit	Immature fruit	Immature pomace
Hesperidin	21,898 ± 108	50,822 ± 662	49,731 ± 808
Narirutin	7,841 ± 92	18,359 ± 275	12,969 ± 165
Sinensetin	12.5 ± 0.9	28.9 ± 1.0	14.5 ± 0.6
Nobiletin	58.8 ± 2.3	138.5 ± 4.9	77.1 ± 1.0
3,5,6,7,8,3′,4′-Heptamethoxyflavone	54.4 ± 3.9	108.7 ± 3.0	69.3 ± 0.8
Tangeretin	41.8 ± 2.1	73.2 ± 2.6	50.9 ± 0.4
Sum	29,908 ± 150	69,530 ± 896	62,912 ± 907

Data are the mean ± SD (n = 3).

### Analyses of single-factor effect experiments

**Figure 1:**
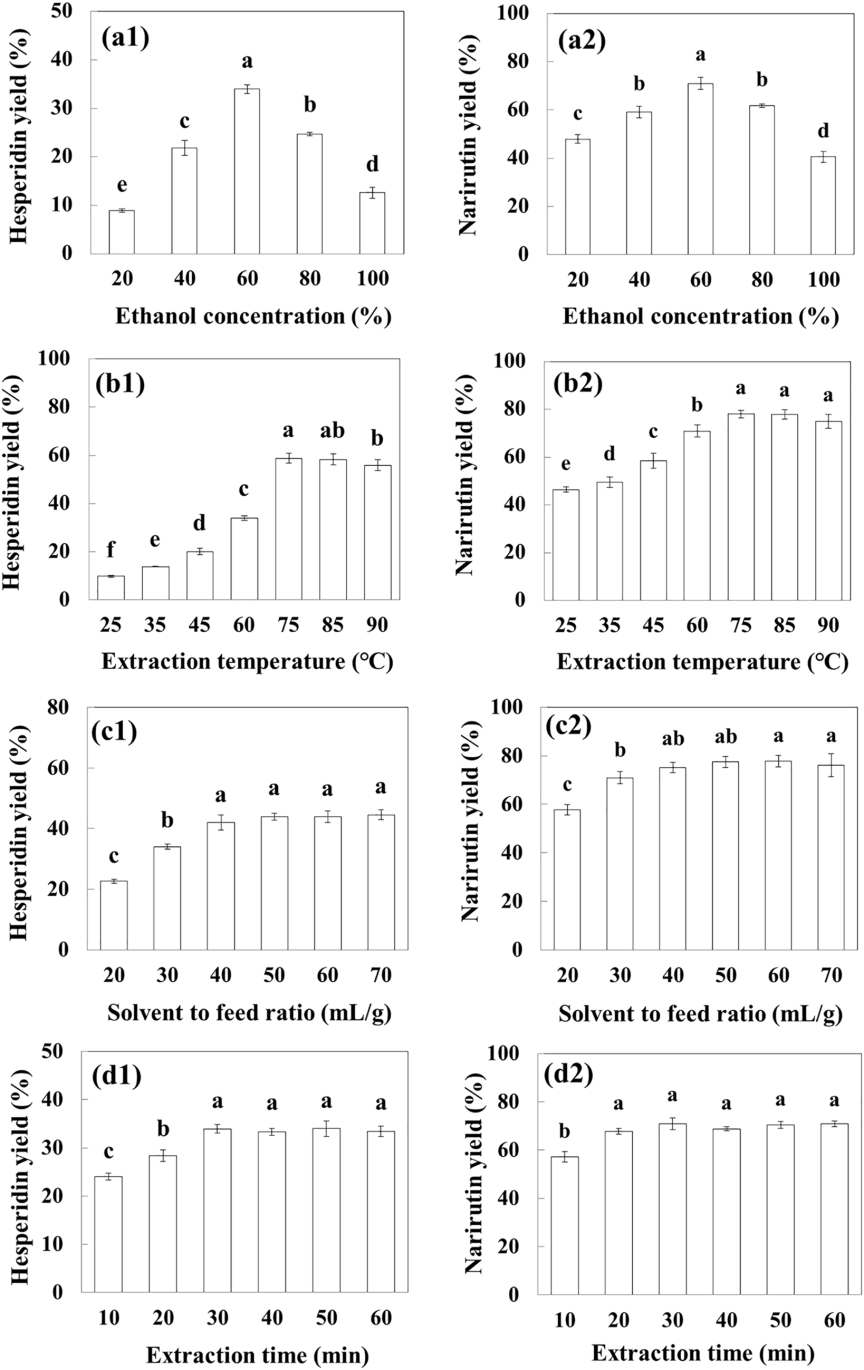
Effects of extraction variables on the extraction yields of hesperidin (**a1**–**d1**) and narirutin (**a2**–**d2**). (**a**) Ethanol concentration, (**b**) temperature, (**c**) solvent to feed ratio, (**d**) extraction time. The extraction yield (%) was calculated as the mass (mg) of each flavonoid in the extract divided by that (mg) in the raw immature *C*. *unshiu* pomace. For each treatment, each bar followed by different letters (^a–e^) is significantly different (n = 3) (*p* < 0.05 using the Duncan’s test).

The effects of extraction parameters (ethanol concentration, temperature, S/F ratio, and extraction time) on the extraction yields of hesperidin and narirutin from ICUP were evaluated individually. The extraction yield (%) was defined as the mass (mg) of each flavonoid in the extract divided by that (mg) in the raw ICUP (Table 2). Figure 1a shows the effects of ethanol concentration (20–100% (v/v)) on the extraction yields of hesperidin and narirutin at 60 °C, 30 mL/g dry sample, and 30 min. The yields of hesperidin and narirutin increased with increasing ethanol concentration from 20 to 60%, but decreased thereafter. Therefore, 40%, 60%, and 80% ethanol were chosen as the RSM working ranges. Generally, the mixture of ethanol and water increases the extraction yields of flavonoids due to the decrease in dielectric constant of the solvent and the increases of solubility and diffusivity of the solute^16,33^. Relatively consistent with this result, a few studies have reported that an approximately 60% ethanol–water mixture is appropriate for phenolic extraction from *C. unshiu* peel^21^ and yuzu (*C. junos* Sieb ex Tanaka) peel^17^. Xu et al.^34^ also reported that the solubility of pure hesperidin in 60% ethanol solution was more than fourfold higher than that in 20% ethanol solution at 60 °C. However, high concentrations of ethanol do not aid extraction due to the dehydration and collapse of plant cells and the denaturation of cell wall proteins^16^.

The effects of temperature (25–90 °C) on the extraction yields of hesperidin and narirutin were measured at 60%, 30 mL/g dry sample, and 30 min (Fig. 1b). The hesperidin yield significantly increased with an increase in temperature from 25 to 75 °C, but slightly decreased at 90 °C. Narirutin showed almost the same trend as hesperidin. Therefore, 60 °C, 75 °C, and 90 °C were chosen as the RSM working ranges. Generally, high temperature increases the extraction yields of flavonoids due to increasing solubility^19^ but decreasing viscosity and surface tension of the solvent^35^. The solubility of pure hesperidin in 60% ethanol–water solution was 1.6-fold higher at 60 °C than at 25 °C^34^. The viscosity of the 63% ethanol–water mixture was 2.313 mPa s at 25 °C, but decreased to 1.108 mPa s at 50°C^36^. However, phenolic compounds can be decomposed and degraded by high temperatures^14^.

The effects of S/F ratio (20–70 g/mL) on the extraction yields of hesperidin and narirutin were also investigated at 60%, 60 °C, and 30 min (Fig. 1c). The yields of both increased with increasing S/F ratio from 20 to 40 mL/g dry sample, after which there were no significantly different effects on the yields (*p* < 0.05). Generally, high S/F ratio increases the extraction yields of flavonoids due to the increase in concentration gradient between the sample and extraction solvent, resulting in increased mass transfer^17,23^. However, a high S/F ratio is economically inefficient due to high-energy consumption in later concentration steps. Therefore, it is important to select the appropriate range of S/F ratio when optimizing the extraction conditions^17,19^. Hence, 20, 30, and 40 mL/g dry sample were chosen as the RSM working ranges.

The effects of extraction time (10–60 min) on the extraction yields of hesperidin and narirutin were determined at 60%, 60 °C, and 30 mL/g dry sample (Fig. 1d). The extraction yields of hesperidin and narirutin were highest at 30 and 20 min, respectively, and there were no significantly different effects on the yields (*p* < 0.05) thereafter. This can also be explained by Fick’s second law of diffusion, which predicts the final equilibrium of the solute concentration between the sample matrix and extraction solvent after a certain period of time^35^. Because an extraction time longer than 30 min is not required for hesperidin or narirutin, the extraction time was fixed at 30 min throughout the optimization process. Iglesias-Carres et al.^19^ reported that an extraction time longer than 30 min did not produce any significant increases or decreases in total phenolic content of the aqueous methanol extract from sweet orange pulp. However, the extraction of hesperidin from *C. unshiu* peel at 1 g/10 mL, 71.5 °C, and 59.0% ethanol needs an extraction time of 12.4 h^21^, and the extraction of hesperidin and naringin from yuzu peel at 1 g/37.1 mL, 43.8 °C, and 65.5% ethanol requires 120 min^17^. Therefore, the optimal conditions for recovering phenolic compounds from citrus may depend on the citrus matrix (peel, pulp, or pomace) used.

## Analyses of RSM experiments

### Response surface optimization

The extraction parameters (extraction temperature, ethanol concentration, and S/F ratio) for the highest yields of hesperidin and narirutin from ICUP were optimized by RSM. Their extraction yields are shown in Table 3, and the results of analysis of variance (ANOVA) of the regression models are presented in Table 4. The regression models for both compounds were a good fit with the experimental data, with low *p*-values (*p* < 0.05), high *R*^2^ values (*R*^2^ ≥ 0.989), nonsignificant lack-of-fit (*p* > 0.05), and low coefficients of variance (CV ≤ 5%)^37^. The following second-order polynomial equations can be used to estimate the optimal conditions for maximizing each flavonoid:

2 $$  \begin{aligned}   {\text{Y (hesperidin) }} &  = {\text{59}}.29 + 8.{\text{88X}}_{1}  + 0.{\text{21X}}_{2}  + 10.{\text{53X}}_{3}  \\     & \quad  - 12.{\text{15X}}_{1}^{2}  - 15.{\text{04X}}_{2}^{2}  - 5.{\text{96X}}_{3}^{2}  - 2.13{\text{X}}_{1} {\text{X}}_{2}  + 1.{\text{70X}}_{1} {\text{X}}_{3}  - 1.{\text{74X}}_{2} {\text{X}}_{3}  \\  \end{aligned} $$

3 $$ \begin{aligned}   {\text{Y (narirutin)}} &  = {\text{77}}.68 + 3.{\text{61X}}_{1}  + 0.{\text{96X}}_{2}  + 7.{\text{76X}}_{3}  \\     & \quad  - 6.{\text{30X}}_{1}^{2}  - 6.{\text{68X}}_{2}^{2}  - 1.{\text{96X}}_{3}^{2}  - 0.{\text{39X}}_{1} {\text{X}}_{2}  - 0.{\text{45X}}_{1} {\text{X}}_{3}  - 1.{\text{92X}}_{2} {\text{X}}_{3}  \\  \end{aligned}   $$

where X_1_, X_2_, and X_3_ are the test variables (temperature, ethanol concentration, and S/F ratio, respectively).

**Table 3:** Box–Behnken design and corresponding hesperidin and narirutin yields from immature *C*. *unshiu* pomace.

Run no.	Uncoded (coded) levels			Extraction yield (%)	
	Temperature (X_1_, ℃)	Ethanol concentration (X_2_, %, v/v)	S/F ratio (X_3_, mL/g dry sample)	Hesperidin	Narirutin
1	60 (− 1)	40 (− 1)	30 (0)	21.8	59.2
2	60 (− 1)	80 (+ 1)	30 (0)	24.7	61.8
3	90 (+ 1)	40 (− 1)	30 (0)	43.8	68.4
4	90 (+ 1)	80 (+ 1)	30 (0)	38.1	69.4
5	60 (− 1)	60 (0)	20 (− 1)	22.6	57.7
6	60 (− 1)	60 (0)	40 (+ 1)	42.0	75.1
7	90 (+ 1)	60 (0)	20 (− 1)	37.0	64.6
8	90 (+ 1)	60 (0)	40 (+ 1)	63.1	80.2
9	75 (0)	40 (− 1)	20 (− 1)	25.7	58.8
10	75 (0)	40 (− 1)	40 (+ 1)	48.6	77.2
11	75 (0)	80 (+ 1)	20 (− 1)	31.5	64.7
12	75 (0)	80 (+ 1)	40 (+ 1)	47.4	75.4
13	75 (0)	60 (0)	30 (0)	56.5	76.9
14	75 (0)	60 (0)	30 (0)	60.0	79.8
15	75 (0)	60 (0)	30 (0)	59.9	77.5
16	75 (0)	60 (0)	30 (0)	60.7	77.4
17	75 (0)	60 (0)	30 (0)	59.3	76.8

The extraction yield (%) was calculated as the mass (mg) of each flavonoid in the extract divided by that (mg) in the raw immature *C*. *unshiu* pomace.

**Table 4:** ANOVA for the regression models.

Source	Hesperidin		Narirutin	
	*F* value	*p*-value	*F* value	*p*-value
Model	117.8	< 0.0001	71.5	< 0.0001
X_1_	193.3	< 0.0001	66.1	< 0.0001
X_2_	0.11	0.7450	4.67	0.0674
X_3_	272.0	< 0.0001	305.7	< 0.0001
X_1_^2^	190.6	< 0.0001	105.8	< 0.0001
X_2_^2^	292.3	< 0.0001	119.2	< 0.0001
X_3_^2^	45.9	0.0003	10.28	0.0149
X_1_X_2_	5.54	0.0508	0.40	0.5453
X_1_X_3_	3.46	0.1011	0.53	0.4900
X_2_X_3_	3.72	0.0953	9.30	0.0186
Lack of fit	1.53	0.3365	1.09	0.4498
*R*^2^	0.993		0.989	
Adj *R*^2^	0.985		0.975	
Pred *R*^2^	0.939		0.913	
%C.V	4.13		1.78	

X_1_: temperature (°C), X_2_: ethanol concentration (%, v/v), X_3_: solvent to feed ratio (mL/g dry sample), pred *R*^2^: predicted *R*^2^, adj *R*^2^: adjusted *R*^2^, C.V.: coefficient of variance.

**Figure 2:**
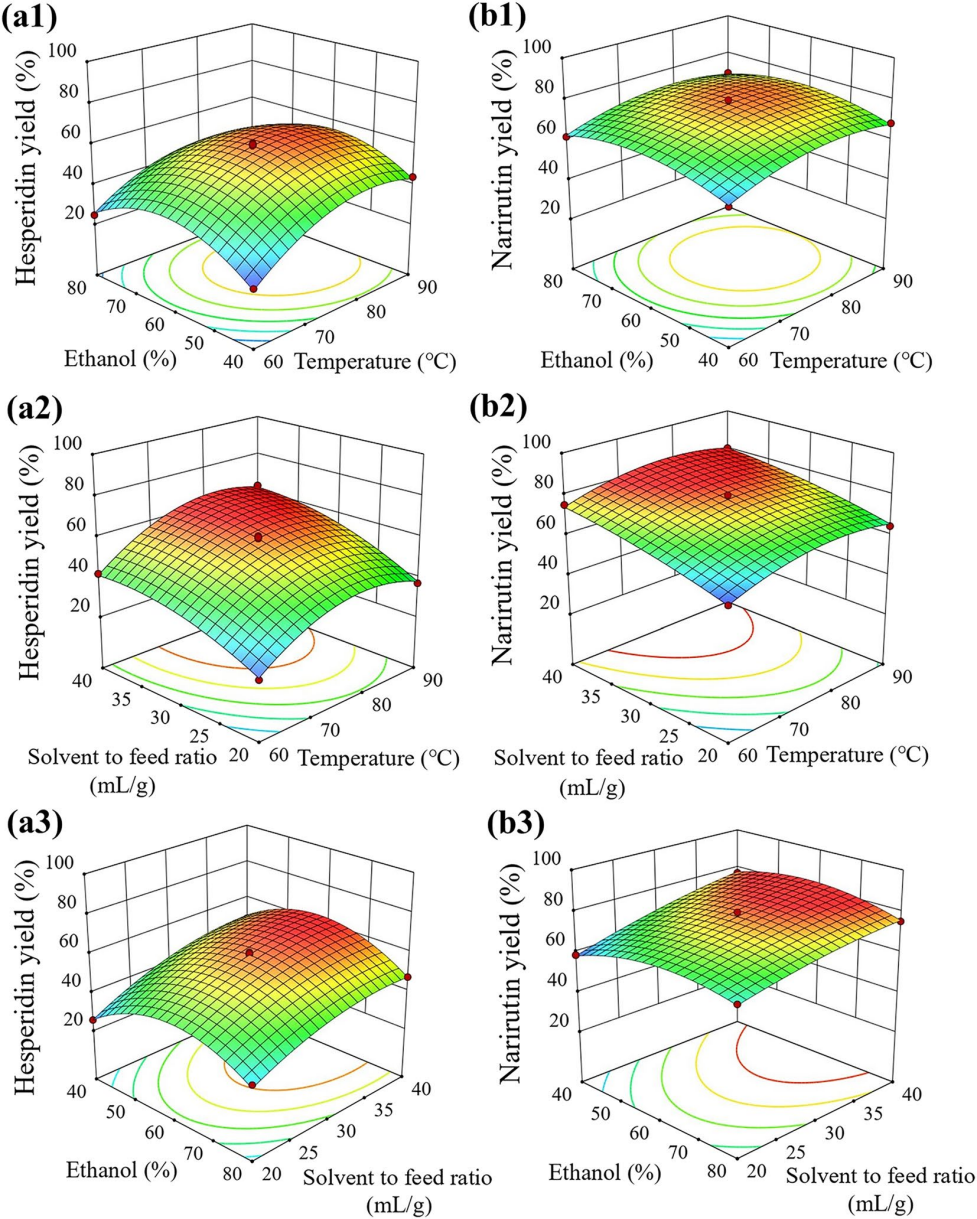
Three-dimensional response surface plots for hesperidin (**a**) and narirutin (**b**) yields.

The regression coefficient for each term in Table 4 indicated the effects of three variables on the extraction yields. The response surface plots also facilitated the visualization of the significance of each extraction variable on the yields (Fig. 2). The positive coefficients of the linear terms (X_1_ and X_3_) and the negative coefficients of the quadratic terms (X_1_^2^ and X_3_^2^) with significant *p*-values (*p* < 0.05) indicated that the yields increased with increases in temperature and S/F ratio, peaking, and then no longer increased with increasing temperature and S/F ratio. A few studies have reported that temperature has positive linear and negative quadratic effects on the optimization of extraction of phenolics from *C. unshiu* peel^21^ and yuzu (*C. junos* Sieb ex Tanaka) peels^17^. On the other hand, the nonsignificant positive linear term (X_2_) and significant negative quadratic term (X_2_^2^) of ethanol concentration indicated that the extraction yield curves of both compounds took the form of a bisymmetry quadratic function with increasing ethanol concentration, and there were maximum ethanol concentrations in both extractions, after which they started to decrease^17,18^. Only the interaction term (X_2_X_3_) between ethanol concentration and S/F ratio for narirutin was significant (*p* < 0.05), indicating that those two parameters significantly affected the yield each other.

Based on the optimized regression models, the optimal extraction conditions at the highest desirability (1.0) were 81.5 °C, 58.4%, and 39.6 mL/g dry sample for hesperidin; and 78.8 °C, 58.4%, and 40.0 mL/g dry sample for narirutin (Table 5). The optimum temperature of hesperidin was 2.7 °C higher than that of narirutin, because hesperidin has a lower solubility in water than narirutin^32^. The optimal extraction conditions at the highest desirability (> 0.977) for simultaneous extraction of hesperidin and narirutin were 80.3 °C, 58.4%, and 40.0 mL/g dry sample, which gave the predicted maximum yields of 66.2% and 83.7% for hesperidin and narirutin, respectively.

**Table 5:** Optimized extraction conditions and predicted yields of hesperidin and narirutin.

Flavanone	Temperature (℃)	Ethanol concentration (%, v/v)	S/F ratio (mL/g dry sample)	Predicted yield (%)	Desirability
Hesperidin	81.5	58.4	39.6	66.3 ± 1.1	1
Narirutin	78.8	58.4	40.0	83.9 ± 0.8	1
Hesperidin + narirutin	80.3	58.4	40.0	Hesperidin: 66.2 ± 1.1 Narirutin: 83.7 ± 0.8	0.977

S/F ratio: solvent to feed ratio (mL/g dry sample). Data are the mean ± SD (n = 3).

### Validation of the model equations

To validate the accuracy of the predicted yields by the model equations, triplicate extractions were performed at the optimal extraction conditions (80.3 °C, 58.4%, and 40 mL/g dry sample) (Table 6). The yields of hesperidin and narirutin at these conditions were 66.6 ± 0.9% and 82.3 ± 1.6%, respectively, in good agreement with the predicted values by the model equations at *p* < 0.05. Therefore, the response models accurately predicted the extraction yields of hesperidin and narirutin within the tested ranges of extraction parameters. Therefore, the temperature, ethanol concentration, and S/F ratio were 80.3 °C, 58.4%, and 40 mL/g dry sample for the rest of the study.

**Table 6:** Predicted and experimental yields of hesperidin and narirutin in the optimum conditions.

Temperature (℃)	Ethanol concentration (%, v/v)	S/F ratio (mL/g dry sample)	Response	Predicted yield (%)	Experimental yield (%)
80.3	58.4	40	Hesperidin	66.2 ± 1.1^a^	66.6 ± 0.9^a^
			Narirutin	83.7 ± 0.8^a^	82.3 ± 1.6^a^

S/F ratio: solvent to feed ratio (mL/g dry sample).

The mean values with the same letter (^a^) in each row are not significantly different (*p* < 0.05 by Student’s *t*-test).

### Number of extractions

**Figure 3:**
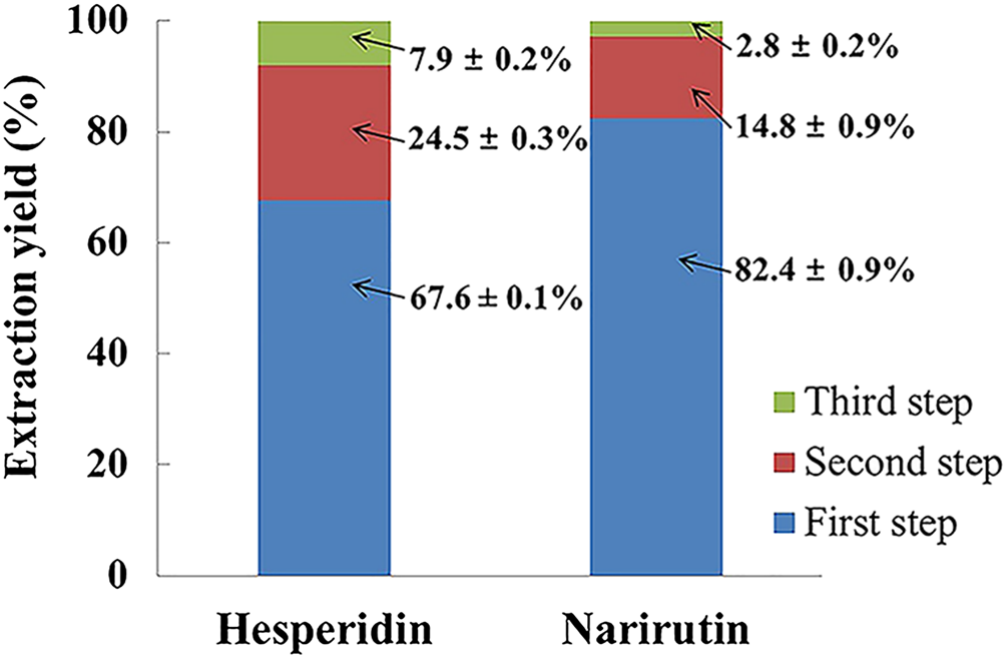
Effect of extraction number on yields of flavanones. Data are the mean ± SD (n = 3).

The effects of the number of extractions were evaluated on the recoveries of each compound from ICUP (Fig. 3). Considerable amounts of hesperidin (67.6%) and narirutin (82.4%) were extracted from the first extraction step and then 24.5% and 14.8% were obtained from the second step, respectively. Because most hesperidin (92.1%) and narirutin (97.2%) were extracted from ICUP in the first two extraction steps and considering practical and economic points, two sequential extractions were proposed as the optimized number of extractions. Iglesias-Carres et al.^19^ reported that 63% and 24% of hesperidin was extracted from *C*. *sinensis* pulp in the first and second extraction steps at the optimum conditions (90% methanol, 55 °C, and 20 mL/g dry sample), respectively.

### Effect of extraction solvent

**Figure 4:**
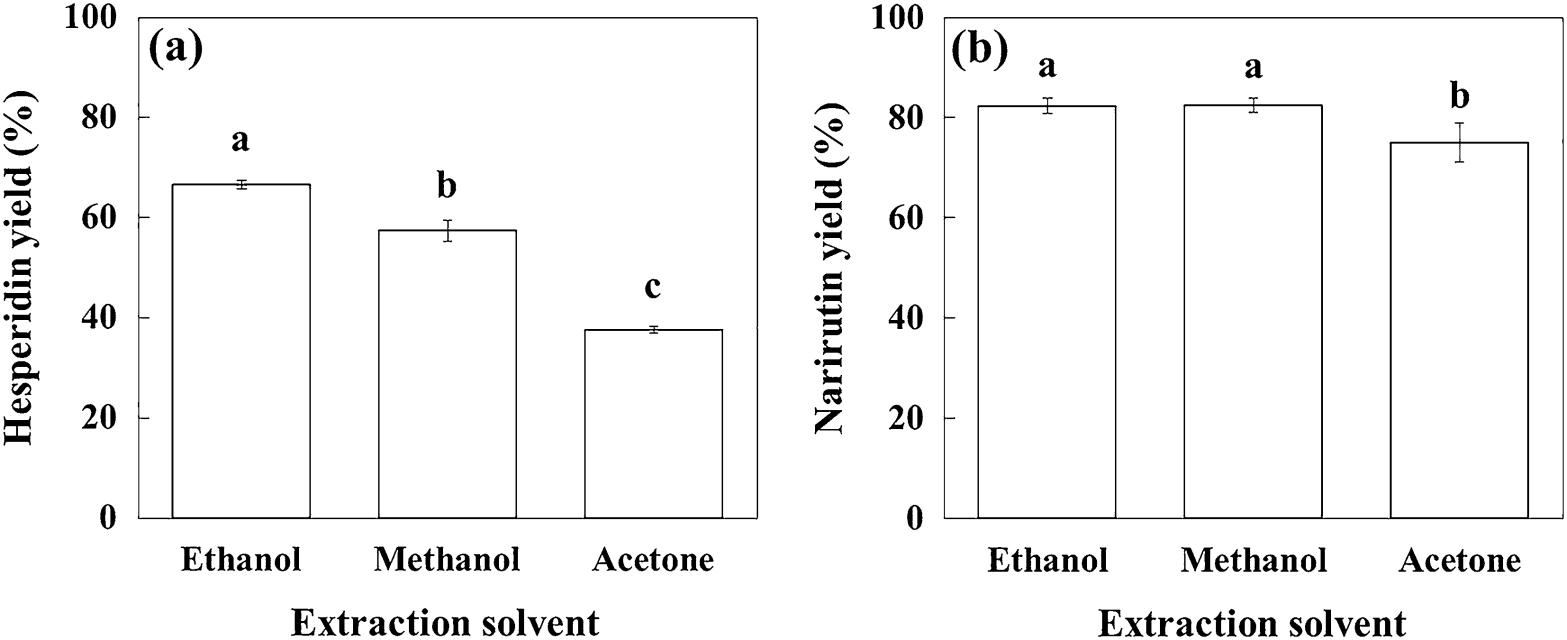
Effect of extraction solvent on yields of flavanones. Each bar followed by different letters (^a–c^) is significantly different (n = 3) (*p* < 0.05 using the Duncan’s test).

To compare the effects of different solvents commonly used for polyphenol extraction on the yields of hesperidin and narirutin, ICUP was extracted using methanol or acetone under the optimized conditions obtained using ethanol (80.3 °C, 58.4%, and 40 mL/g dry sample for 30 min) (Fig. 4). The yield of hesperidin was highest at 66.6% in ethanol, followed by 57.3% in methanol and 37.7% in acetone. The extraction yield of narirutin in ethanol (82.3%) was statistically the same as that in methanol (82.5%), and that in acetone was the lowest (75.1%). Ethanol was more effective for extracting hesperidin and narirutin than methanol and acetone. Generally, ethanol is a suitable extraction solvent for flavonoids and their glycosides, catechol, and tannin; methanol for phenolic acid and catechin; and acetone for high-molecular-weight polyphenols such as proanthocyanidins and tannins^35^.

In some other studies, higher extraction yields of hesperidin were obtained using methanol than ethanol. Magwaza et al.^38^ reported that the yield of hesperidin from mandarin (*C*. *reticulata*) rinds was higher in 70% methanol than in 80% ethanol at 35 °C. Iglesias-Carres et al.^19^ also observed that the hesperidin yield from *C*. *sinensis* pulp was higher in 90% methanol than in 90% ethanol at 55 °C. These differences are thought to be due to differences in the extraction temperature and ethanol concentration. In most extraction studies using ethanol as the solvent, the optimal extraction temperature was 48–95 °C, and the optimal ethanol concentration was 51–59% for phenolic and flavonoid compounds from kaffir lime peels^39^, *C*. *unshiu* peels^21^, and yuzu peels^17^.

### Antioxidant activity

The antioxidant activities of natural antioxidants from plant materials depend on the reaction mechanism based on the multiplicity and heterogeneity of the matrix and the distribution of antioxidant compounds between the lipophilic and hydrophilic phases. Therefore, the antioxidant activities of plant extracts cannot be properly assessed by only one method^40,41^. In this work, 10 assay methods were used to comprehensively evaluate the antioxidant activities of ethanol, methanol, and acetone extracts: radical scavenging activities (DPPH, ABTS, nitric oxide, hydroxyl, superoxide anion, and ORAC), scavenging activities of nitrite and hydrogen peroxide, and reducing capacity (FRAP and reducing power) (Table 7).

**Table 7:** Antioxidant activities of ethanol, methanol, and acetone extracts from immature *C*. *unshiu* pomace.

Extraction solvent	Antioxidant activity (mg Trolox equivalents/g dry sample)									
	Nitrogen radicals		RNS		ROS				Reducing abilities	
	DPPH	ABTS	Nitrite (NO_2_^−^)	Nitric oxide (NO∙)	ORAC (ROO∙)	Hydroxyl radical (∙OH)	Superoxide anion (∙O_2_^-^)	Hydrogen peroxide (H_2_O_2_)	Reducing power	FRAP
Ethanol	4.4 ± 0.2^a^	17.9 ± 0.3^a^	17.9 ± 0.6^a^	4.2 ± 0.1^a^	237.5 ± 8.8^a^	206.1 ± 11.3^a^	394.5 ± 13.3^a^	74.5 ± 1.7^a^	7.8 ± 0.2^a^	14.5 ± 0.8^a^
Methanol	3.5 ± 0.1^b^	16.5 ± 0.3^b^	15.1 ± 0.8^b^	3.4 ± 0.2^b^	197.6 ± 8.7^b^	128.1 ± 9.4^b^	327.8 ± 10.8^b^	69.8 ± 2.0^a^	7.2 ± 0.3^b^	12.9 ± 0.5^b^
Acetone	2.9 ± 0.1^c^	15.0 ± 0.2^c^	9.8 ± 0.3^c^	3.0 ± 0.1^c^	174.8 ± 11.1^c^	77.0 ± 5.9^c^	235.6 ± 5.5^c^	57.3 ± 3.2^b^	7.2 ± 0.2^b^	11.6 ± 0.3^c^

The mean values with different letters (^a–c^) in each column are significantly different (*p* < 0.05 by Duncan test).

*RNS* reactive nitrogen species, *ROS* reactive oxygen species.

DPPH and ABTS radical scavenging activities are commonly used to measure the antioxidant activities of natural antioxidants due to the stability of nitrogen radicals and the simple method used to measure these^42^. DPPH assays use radicals dissolved in organic solvents, which is applicable to the hydrophobic system, but the ABTS assay is applicable to both hydrophilic and lipophilic systems^43^. DPPH and ABTS radical scavenging activities were higher in the ethanol extract than in methanol and acetone extracts. The ABTS radical scavenging activity was 4.06-fold higher than the DPPH radical scavenging activity in the ethanol extract, indicating that its radical scavenging activity works better in a hydrophilic system.

RNS and ROS are directly involved in oxidative stress and are closely correlated with the development of several human diseases including atherosclerosis, diabetes, chronic inflammation, and neurodegenerative disorders^3^. Nitrite and nitric oxide radicals are representative RNS. RNS scavenging activity was higher in the ethanol extract than in methanol and acetone extracts. The nitrite scavenging activity was 4.26-fold higher than nitric oxide radical scavenging activity in the ethanol extract. ROS scavenging activities (ORAC, hydroxyl radical, and superoxide anion radical) were higher in the ethanol extract than in methanol and acetone extracts, but there were no differences in hydrogen peroxide scavenging activity between ethanol and methanol extracts. The ethanol extract showed very high ROS scavenging activities (ORAC: 237.5, hydroxyl radical: 206.1, superoxide anion radical: 394.5, and hydrogen peroxide: 74.5 mg Trolox equivalents (TE)/g dry sample) compared to RNS scavenging activities (nitrite: 17.9, nitric oxide: 4.2 mg TE/g dry sample). Most ROS have a short half-life and a high concentration of ROS induces oxidative damage to lipids, proteins, and DNA^44,45^. Therefore, the strong and rapid ROS scavenging properties of the ethanol extract can actually prevent the oxidation of food and help fight human diseases caused by ROS.

The reducing capacity of each extract was also measured using two methods: a reducing power assay and an FRAP assay. Both methods are commonly used to measure the reducing power of natural antioxidants^45^. The reducing power and FRAP were higher in the ethanol extract than in the methanol and acetone extracts. The FRAP was about 1.85-fold higher than reducing power in the ethanol extract. This difference between two methods may be due to their different reducing mechanisms. In FRAP, the antioxidant reduces Fe^3+^ to Fe^2+^ in the Fe–TPTZ complex^46^, whereas in reducing power, the antioxidant reduces ferricyanide to ferrocyanide^47^.

The ethanol extract showed very high scavenging activities against the radicals containing only oxygen (ROO∙ and ∙O_2_^−^) or oxygen with hydrogen (∙OH and H_2_O_2_), but relatively low scavenging activities against nitrogen radicals (DPPH∙ and ABTS∙) and nitrogen containing radicals (NO_2_^−^ and NO∙), and relatively low reducing activities against the Fe-complex containing nitrogen (Fe(CN)_6_^3−^ and [Fe(III)(TPTZ)_2_]^3+^). These results indicate that the antioxidant activity of the extract from ICUP may vary depending on the structure of the radicals, the reaction mechanisms, and the flavonoid types.

Table 8 shows the Pearson correlation coefficients representing the relationship between antioxidant activities and the contents of hesperidin and narirutin in ethanol, methanol, and acetone extracts. The hesperidin content showed a higher correlation (0.923–1.000) with all of the antioxidant activities than the narirutin content (0.741–0.958) except the reducing power, which means that the hesperidin in the extracts had a significant effect on antioxidant activities due to its high content and strong antioxidant activity. M’hiri et al.^48,49^ also reported that hesperidin had higher antioxidant activity than narirutin due to the presence of a catechol group in the B-ring of the hesperidin molecule.

**Table 8:** Pearson correlation coefficients between antioxidant activities and flavonoid contents in the extracts.

Flavanone	DPPH	ABTS	Nitrite (NO_2_^−^)	Nitric oxide (NO∙)	ORAC (ROO∙)	Hydroxyl radical (∙OH)	Superoxide anion (∙O_2_^−^)	H_2_O_2_	Reducing power	FRAP
Hesperidin + narirutin	0.941	0.978	0.999	0.913	0.927	0.940	0.991	1.000	0.730	0.959
Hesperidin	0.949	0.983	1.000	0.923	0.935	0.947	0.994	0.999	0.746	0.965
Narirutin	0.789	0.865	0.932	0.741	0.764	0.787	0.899	0.958	0.481	0.822

## Conclusions

We optimized the extraction method for the highest recoveries of hesperidin and narirutin from ICUP, and the optimal extraction conditions were two sequential extractions at a temperature of 80.3 °C, ethanol concentration of 58.4% (v/v), and S/F ratio of 40 mL/g dry sample with a 30 min extraction time, where the hesperidin and narirutin yields were 92.1% and 97.2%, respectively. The ethanol extract showed higher antioxidant activities measured using nine different assay methods than methanol and acetone extracts. The ethanol extract showed very high scavenging activities against ROS. Hesperidin contents in ethanol, methanol, and acetone extracts showed a higher correlation with all of the antioxidant activities except the reducing power than narirutin contents. Therefore, the ethanol extract of immature *C*. *unshiu* pomace could be used as a flavonoid supplement for preventing human diseases caused by ROS. Further research is recommended to verify the anti-aging effect of those extracts based on experiments with cells and animals.

## Supplementary information

Supplementary Information.
