# Supplementary material for: Extraction of flavanones from immature Citrus unshiu pomace: process optimization and antioxidant evaluation
Source: Sci Rep. 2020 Nov 17;10:19950. doi: 10.1038/s41598-020-76965-8 (PMC7673033; doi:10.1038/s41598-020-76965-8)
Supplement: Supplementary file 1 — Supplementary Information. [file 41598_2020_76965_MOESM1_ESM.docx]

**Extraction of flavanones from immature *Citrus unshiu* pomace: process optimization and antioxidant evaluation**

Dong-Shin Kim & Sang-Bin Lim^*^

**Supplementary Materials**

**Chemicals and reagents**

Hesperidin (94%), (±)-6-hydroxy-2,5,7,8-tetramethylchromane-2-carboxylic acid (Trolox), 2,2′-azobis(2-methylpropionamidine) dihydrochloride (AAPH), 2,2′-azino-bis(3-ethylbenzothiazoline-6-sulfonic acid) diammonium salt (ABTS), 2,2-diphenyl-1-picrylhydrazyl (DPPH), fluorescein sodium salt, Griess reagent, sodium nitroprusside, N-(1-naphthyl)ethylenediamine dihydrochloride, hydrogen peroxide (H_2_O_2_), 1,10-phenanthroline, iron(II) sulfate heptahydrate (FeSO_4_∙7H_2_O), 2,4,6-tris(2-pyridyl)-s-triazine (TPTZ), β-nicotinamide adenine dinucleotide (NADH), and potassium ferricyanide, ferric chloride (FeCl_3_) were obtained from Sigma-Aldrich (St. Louis, MO, USA). Sulphanilamide, ρ-nitroblue tetrazolium chloride, and phenazine methosulphate were obtained from Tokyo Chemical Industry Co., Ltd. (Tokyo, Japan). Narirutin (98%) was purchased from Avention (Incheon, Korea), and sodium nitrite was obtained from Fujifilm Wako Pure Chemical Corp. (Osaka, Japan). Methanol (99.9%) and ethanol (99.8%) were obtained from Carlo Erba Reagents (Val de Reuil, France), and acetone (99.5%) was purchased from TEDIA (Fair-field, OH, USA). Potassium persulfate and acetonitrile (HPLC grade) were obtained from Daejung Chemicals & Metals Co., Ltd (Gyeonggi, Korea).
